# The anterior cruciate ligament in murine post-traumatic osteoarthritis: markers and mechanics

**DOI:** 10.1186/s13075-022-02798-7

**Published:** 2022-05-30

**Authors:** Lorenzo Ramos-Mucci, Ahmed Elsheikh, Craig Keenan, Ashkan Eliasy, Kristiaan D’Aout, George Bou-Gharios, Eithne Comerford, Blandine Poulet

**Affiliations:** 1 Institute of Life Course and Medical Sciences, University of Liverpool, Apex building, West Derby street, Liverpool, L7 8TX UK; 2 School of Engineering, University of Liverpool, Brownlow Hill, Liverpool, L69 3GH UK; 3 Beijing Advanced Innovation Centre for Biomedical Engineering, Beihang University, Beijing, 100083 China; 4 NIHR Biomedical Research Centre for Ophthalmology, Moorfields Eye Hospital NHS Foundation Trust and UCL Institute of Ophthalmology, London, UK; 5 Faculty of Health, Social Care and Medicine, Edge Hill University, St Helens Road, Ormskirk, Lancashire L39 4QP UK; 6 School of Veterinary Science, Institute of Infection, Veterinary and Ecological Sciences, Leahurst Campus, University of Liverpool, Chester High Rd, Neston, CH64 7TE UK

**Keywords:** Anterior cruciate ligament, Mechanics, Viscoelastic, Chondrogenesis, Post-traumatic osteoarthritis

## Abstract

**Background:**

Knee joint injuries, common in athletes, have a high risk of developing post-traumatic osteoarthritis (PTOA). Ligaments, matrix-rich connective tissues, play important mechanical functions stabilising the knee joint, and yet their role post-trauma is not understood. Recent studies have shown that ligament extracellular matrix structure is compromised in the early stages of spontaneous osteoarthritis (OA) and PTOA, but it remains unclear how ligament matrix pathology affects ligament mechanical function. In this study, we aim to investigate both structural and mechanical changes in the anterior cruciate ligament (ACL) in a mouse model of knee trauma.

**Methods:**

Knee joints were analysed following non-invasive mechanical loading in male C57BL/6 J mice (10-week-old). Knee joints were analysed for joint space mineralisation to evaluate OA progression, and the ACLs were assessed with histology and mechanical testing.

**Results:**

Joints with PTOA had a 33–46% increase in joint space mineralisation, indicating OA progression. Post-trauma ACLs exhibited extracellular matrix modifications, including COL2 and proteoglycan deposition. Additional changes included cells expressing chondrogenic markers (SOX9 and RUNX2) expanding from the ACL tibial enthesis to the mid-substance. Viscoelastic and mechanical changes in the ACLs from post-trauma knee joints included a 20–21% decrease in tangent modulus at 2 MPa of stress, a decrease in strain rate sensitivity at higher strain rates and an increase in relaxation during stress-relaxation, but no changes to hysteresis and ultimate load to failure were observed.

**Conclusions:**

These results demonstrate that ACL pathology and viscoelastic function are compromised in the post-trauma knee joint and reveal an important role of viscoelastic mechanical properties for ligament and potentially knee joint health.

**Supplementary Information:**

The online version contains supplementary material available at 10.1186/s13075-022-02798-7.

## Introduction

Osteoarthritis (OA) is a degenerative disease that affects the whole joint [1], including loss of articular cartilage, subchondral bone remodelling, synovial hyperplasia and ligament degeneration [1]. Ligaments are the main stabilisers of joints [2], and injury often leads to post-traumatic OA (PTOA) development. Their mechanical and cellular properties following trauma, however, are largely unexplored. Previously, we showed structural and pathological changes in the cruciate and collateral knee ligaments of spontaneous OA and PTOA mouse models [3], similar to the pathology described in human OA anterior cruciate ligaments (ACLs) [4, 5]. These pathological changes could alter ligament mechanical function and consequently knee stability and OA progression. In this study, we aimed to investigate both structural and mechanical changes in the ACL in a PTOA model.

Ligament composition is closely associated to its mechanical function [2]. Ligaments are composed of a collagen hierarchical structure made primarily of collagen type I and of non-collagenous extracellular matrix (ECM) proteins such as proteoglycans, elastin and glycolipids [2]. Ligaments are viscoelastic and demonstrate (1) strain rate sensitivity, where mechanical behaviour is stiffer at higher strain rates, (2) stress-relaxation, where stress reduces under constant deformation, (3) creep, where deformation occurs under a constant load and (4) hysteresis, where loading and unloading behaviour differ [6]. The structural components that control viscoelastic behaviour are still debated. Of interest are glycosaminoglycan chains found in proteoglycans which interact with collagen fibrils and determine stiffness [7]. In addition, proteoglycan deficiency can alter tendon viscoelastic behaviour [8–10]. Other ECM proteins involved with viscoelasticity include collagen V [11, 12] and elastin [13].

The ACL is the most commonly injured knee ligament and injury to the ACL is associated with PTOA development [14]. After injury, there is an acute release of matrix components, proteases and cytokines from cartilage and surrounding joint tissues [14]. Furthermore, an injured ACL results in abnormal loading patterns, including abnormal anterior tibial translation, changes to tibial rotation, changes to tibiofemoral contact patterns [15] and increased forces to the cartilage and menisci [14]. ACL tears can occur both at the mid-substance (higher prevalence) or enthesis resulting in avulsion fractures [16]; therefore, understanding ACL microanatomical changes following trauma can be clinically important to address different types of ACL tears.

To date, there are few studies that characterise ACL mechanics in post-traumatic or spontaneous OA. In late-stage human OA patients, one study found decreased elastic stiffness and increased viscoelastic stress-relaxation in ACLs [17]. Animal models of OA show similar pathways to human OA and include spontaneous and mechanical induction models [18]. In Dunkin Hartley guinea-pig spontaneous OA model, the viscoelastic toe-region laxity was reduced [19]. ACLs from osteoarthritic STR/ort mice had lower ultimate load compared to healthy joints [20]. These studies confirm a decrease in stiffness and ultimate load in the ACL of age-related osteoarthritic joints, but ACL mechanics following trauma remains uncharacterised, particularly viscoelastic properties which are physiologically relevant [21].

In this study, we used an established non-invasive model of PTOA, which replicates knee trauma and PTOA development similar to humans [22–25] and allows for testing of the ACL. We hypothesised that changes in the ACL ECM composition and structure following trauma will result in reduced stiffness and modifications of their viscoelastic properties. We have tested this by characterising structural changes and mechanical properties of the ACL after trauma. This research will describe ECM and cellular pathology and mechanics following trauma. This is necessary for the development of future therapeutical interventions.

## Materials and methods

### Post-traumatic osteoarthritis (PTOA) mouse model

Non-invasive joint loading was performed on the right knees of 10-week-old C57BL/6 male mice (Charles River, UK) to induce progressive PTOA, as previously reported [22]. Briefly, 9N axial compressive loads were applied for forty cycles (ElectroForce 3100, TA Instruments, USA), with six loading episodes over a period of 2 weeks. Order of treatment was randomised. Mice were kept under general gas anaesthesia (isoflurane) for the entire procedure. Knee joints were analysed at 4 and 14 weeks post-trauma for joint mineralisation and histology and at 6 weeks post-trauma for ACL mechanics and compared to aged-matched healthy knee joints. At 6 weeks post-trauma, PTOA progression in this model has been previously described and includes significant loss of hyaline articular cartilage in the lateral femur [24]. This indicates early stages of PTOA development at 6 weeks post-trauma.

All mice were kept in the same conditions in polypropylene cages, subjected to 12-hour light/dark cycles, at 21 ± 2 °C and fed standard diet ad libitum. Animals were euthanised by cervical dislocation and tissue samples collected and treatment group blinded. All procedures were performed according to UK Home Office guidelines and regulations under the Animals (Scientific Procedures) Act 1986 and local ethics committee (project license: P267B91C3).

### Measuring knee joint space mineralisation in the PTOA knee joint

For joint space mineralisation, 4 knee joint conditions were compared: aged control + 4 weeks (*n* = 4), aged control + 14-weeks (*n* = 4), post-trauma + 4-weeks (*n* = 7), and post-trauma + 14-weeks (*n* = 4). *N* = 4 provided > 80% power when comparing between healthy and PTOA knee joints due to a high difference in population means (Power and Sample Size Software, version 3.1.6, Vanderbilt).

Knee joint mineralisation is a new method associated with OA progression and increases progressively in post-traumatic and spontaneous OA mouse models [3]. Cadaveric knee joints were fixed (neutral buffered formalin), dehydrated and scanned using a Skyscan 1172 μCT scanner (Skyscan, Belgium) with a 5-μm isotropic voxel size (50 kV, 200 μA, 0.5 mm Aluminium filter, 0.6° rotation angle, no frame averaging). Regions of interests of the knee joint comprising of menisci and ligaments were hand-drawn and quantified using CTAn (Skyscan, Belgium) [3]. Statistical analysis of mineralised volume of the knee joint was performed using a one-way analysis of variance and post-hoc analysis to compare control and PTOA knee joints (significance was set at *p* < 0.05). Three-dimensional models of the mineralised tissue were created using CTVox (Skyscan, Belgium).

### Histological and immunohistochemical assessment of the mouse knee joint

For ACL histology, knee joints (*n* = 6) were analysed at + 6 weeks post-trauma and compared to age-matched controls (*n* = 6). Fixed (neutral buffered formalin) joints were decalcified (ImmunocalTM, Quarttet, Berlin, Germany), dehydrated and processed for wax embedding. Serial coronal 6-μm-thick sections were stained with toluidine blue (TB) to assess pathophysiological changes in the knee joint [26] and picrosirius red to assess collagen birefringence [27]. For detailed staining protocols, see Supplementary Information 10.1.

Immunohistochemistry was performed to localise expression of collagen type II (COL2) (Thermo, MA5-12789, Mouse monoclonal), SOX9 (Millipore, AB5535, Rabbit polyclonal), RUNX2 (Abcam, ab23981, Rabbit polyclonal) and asporin (ASPN) (Abcam, ab58741, Rabbit polyclonal), similar to previously published protocols [3]. For detailed protocols, see Supplementary Information 10.2.

### Anterior cruciate ligament (ACL) imaging and measurements

**Fig. 1:**
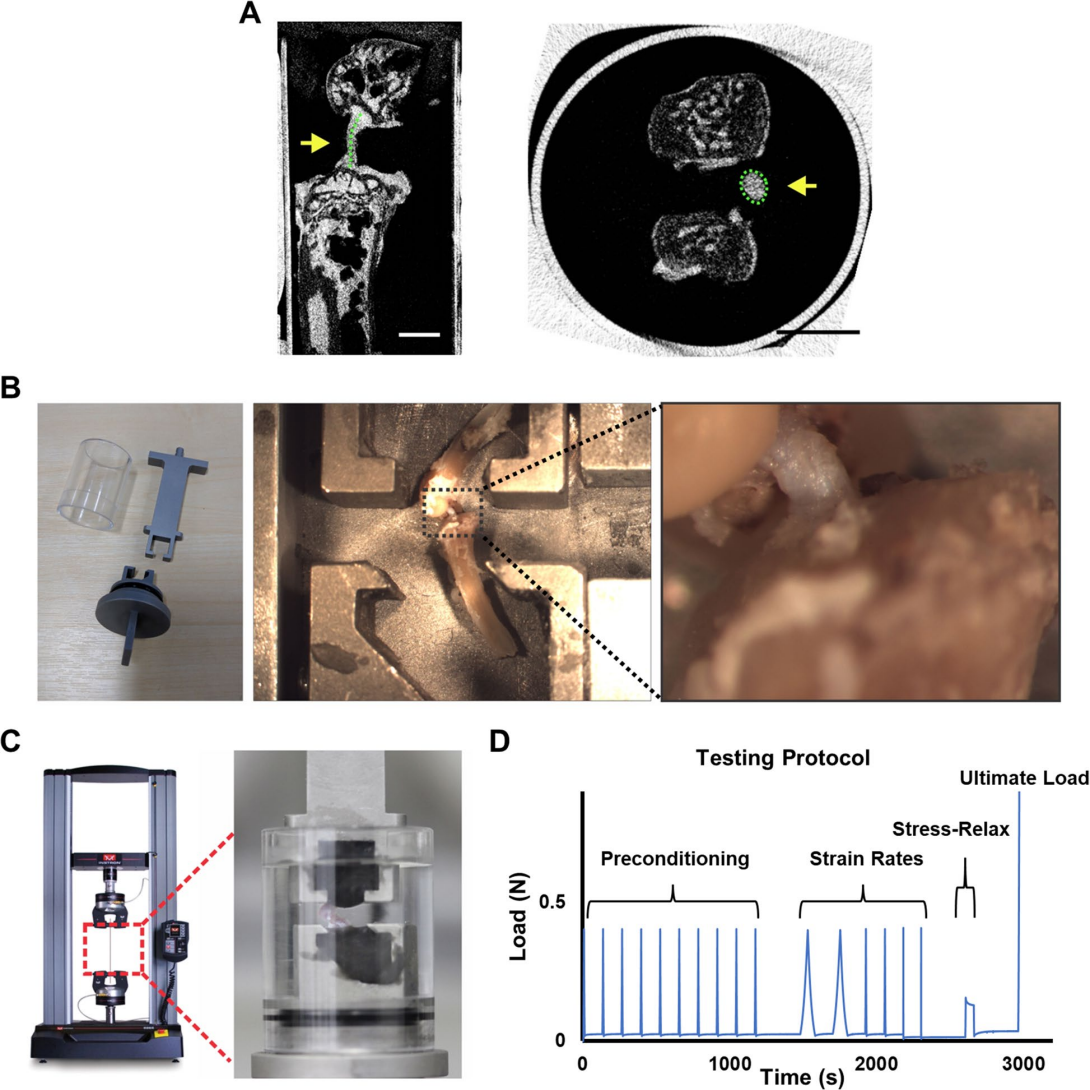
Murine anterior cruciate ligament (ACL) imaging and mechanical testing setup. **A** The ACL was imaged with μCT using a contrast agent. ACL length and cross-sectional area (CSA) (yellow arrows) were measured. Scale bar is 1 mm. **B** Mechanical testing setup included custom-made clamps, which allowed for poly(methyl methacrylate) fixation of the femur and tibia at a 90° angle and for vertical alignment of the ACL. **C** Fixed samples were loaded into a dual column uniaxial machine (Instron) for mechanical testing of the ACL. **D** Mechanical testing protocol used for viscoelastic and material behaviour of the ACL included preconditioning cycles, strain rate testing at 0.1%/s, 1%/s and 10%/s strain rates, stress-relaxation loaded to 5% strain and ultimate load to failure at 1%/s until ACL rupture

ACL length and cross-sectional area (CSA) were measured at + 6 weeks post-trauma using μCT imaging (Fig. 1A). ACLs from the right knee joint following trauma (*n* = 4) were compared to healthy ACL samples from the contra-lateral left knee joint (*n* = 4). It has been shown that contralateral ACLs are an appropriate surrogate for anatomical dimensions [28]. Hindlimbs were kept at − 20 °C until used for analysis. Samples were defrosted and dissected under a microscope until only the femur-ACL-tibia complex remained. Femur-ACL-tibia samples were submerged in Lipiodol contrast agent (Guerbet, FR) overnight at 4 °C. Samples were fitted into a straw filled with Lipiodol, and a putty adhesive (Blu Tack) kept the femur at a 90° angle with the ACL and tibia suspended vertically. The femur-ACL-tibia samples were then scanned with a 5-μm isotropic voxel size (50 kV, 200 μA respectively, 0.5 mm Aluminium filter; 0.6° rotation angle, no frame averaging) using a Skyscan 1172 μCT scanner (Bruker, BE), as previously described [29].

A sagittal image, showing the femoral condyles-ACL-and tibia condyles, was used to measure the ACL length using CTan software (Bruker) (Fig. 1A, yellow arrow). A second area of interest of the entire ACL was analysed in the axial plane for analysis of the ACL CSA. CSA was measured using CTan software (Bruker) at the ACL midpoint (Fig. 1A, yellow arrow). An average ACL length and CSA was calculated for each mouse group and used to calculate stress-strain behaviour.

### Mechanical testing of ACLs

For ACL mechanical testing, knee joints were analysed at + 6 weeks post-trauma. Post-trauma ACLs (right knee joints, *n* = 7) were compared to aged-matched healthy ACLs (right knee joint, *n* = 7). Power analysis was conducted with preliminary data of ACL tangent modulus calculations using Power and Sample Size Software (version 3.1.6, Vanderbilt). *N* = 7 was greater than the ideal sample size needed (*n* = 6) to detect a 15% difference between post-trauma and control population means.

ACL mechanical properties were analysed with a customised clamp and tensile testing system (Fig. 1B), which was designed based on past literature [11, 12, 20, 30].

Mouse hindlimbs were stored at − 20 °C. This storage temperature does not influence the viscoelastic and mechanical properties of ligaments [31, 32]. On the day of measurement, knees were defrosted and dissected until only the femur-ACL-tibia complex remained. Poly(methyl methacrylate) (Technovit 6091) was used to fix the femur and tibia, to approximate 90° of knee flexion (Fig. 1B). 90° was within the physiological range of mouse knee flexion (Sup. Table 1) and allowed for tensile testing along the axis of the ligament [30].

The femur-ACL-tibia was briefly aligned under the dissection microscope to ensure a vertical ACL orientation (Fig. 1B). The sample was transferred to a dual column uniaxial material testing machine (Instron 3366, USA) with a 10N load cell. The encasing outer covering enclosed the sample and phosphate-buffered saline was added to ensure a hydrated ligament (Fig. 1C).

The mechanical testing protocol applied a preload of 0.02 N to remove laxity in the ligament, followed by a series of 10 preconditioning cycles at a strain rate of 1%/s to a maximum load of 0.4N [33] (Fig. 1D). Following preconditioning, strain rate sensitivity, stress-relaxation behaviour and ultimate load at failure were tested (Fig. 1D). For strain rate testing, two load-unload cycles of three different strain rates were applied (0.1%/s, 1%/s, and 10%/s), as previously reported [12, 20]. Viscoelastic stress-relaxation was tested at 5% strain (applied strain at 1%/s) and elongation was maintained while monitoring load [11]. Elongation was maintained for 120 s where stress-relaxation behaviour started to stabilise (Fig. 1D). Lastly, the ultimate load at failure was tested by increasing the load on the specimen while maintaining a strain rate of 1%/s until ligament rupture took place (Fig. 1D).

Analyses of the load-elongation data were performed on Microsoft Excel (version 2109). Stress, strain tangent modulus and hysteresis values were calculated as previously described [34, 35]. For calculations of each viscoelastic property, see Supplementary Section 10.3.

### Statistical analysis

For statistical analysis of the ACL viscoelastic properties (stress-strain, tangent modulus, strain rate sensitivity, normalised strain rate sensitivity), measurements were analysed at specified stress or strains. For stress-relaxation, the area under the curve was calculated using numerical integration and used to compare control and post-trauma ACL.

Statistical analysis was calculated using GraphPad Prism (v8, GraphPad, USA). Data normality was assessed with Shapiro–Wilk tests. For ACL CSA and length measurements, an unpaired *t*-test was used to compare between control and test groups. Data was not normally distributed for the stress-strain of the post-trauma and control group, and therefore, a nonparametric statistical test was performed (Mann-Whitney test). A nonparametric Friedman test was used when comparing the stress-strain at the three different strain rates, followed by a Dunn’s multiple comparison test. All other ACL properties showed normal distribution and an unpaired *t*-test (two tailed) was performed to compare control and test groups. A repeated measures analysis of variance was used when comparing the tangent modulus-stress and hysteresis at different strain rates, followed by a Bonferroni post-hoc test. For stress-relaxation, when comparing the normalised tangent modulus of the 1%/s and the 10%/s strain rates, a paired *t*-test (two-tailed) was used. A Friedman test, repeated measures analysis of variance and paired *t*-test were used since measurements were taken from the same sample at different strain rates. Significance was set at *p* < 0.05.

## Results

### Knee joint space mineralisation increased in PTOA knee joints

**Fig. 2:**
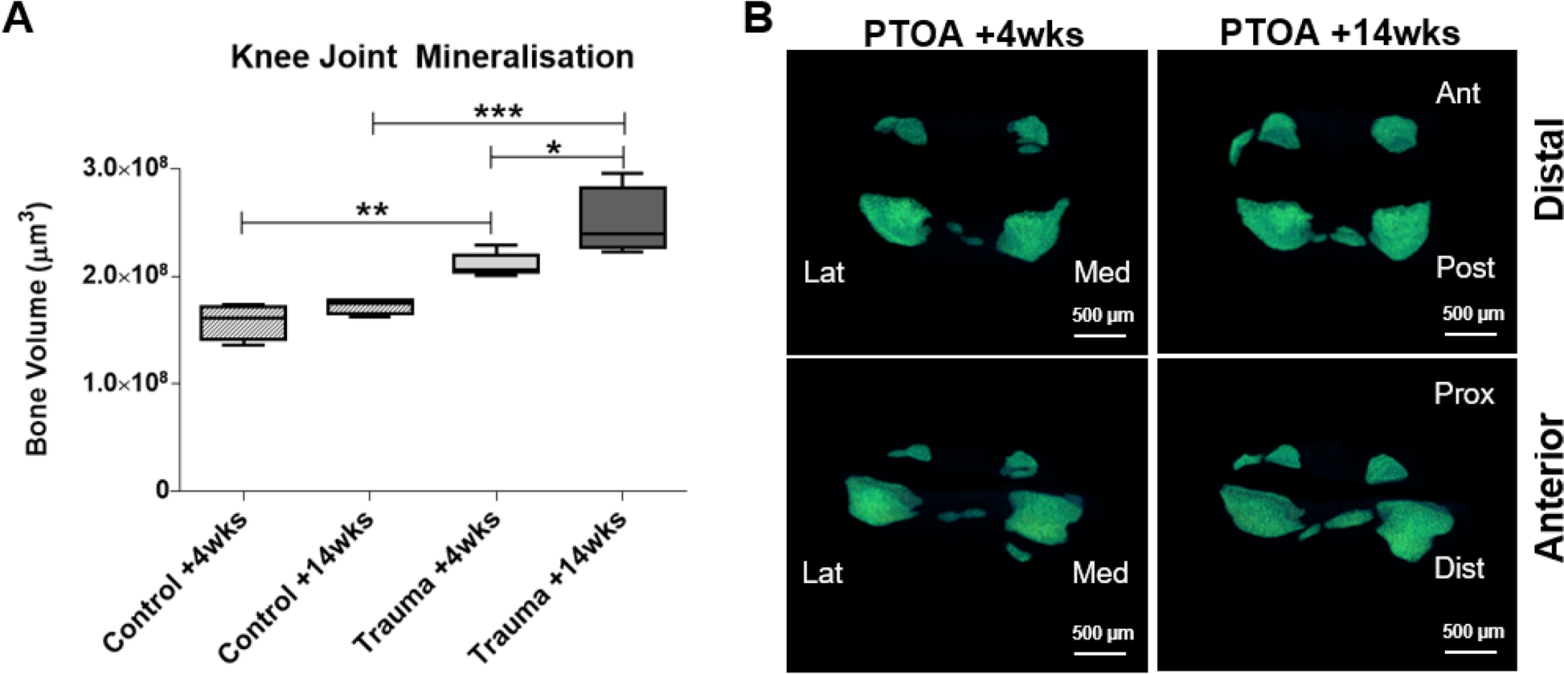
Quantification and 3D images of joint space mineralisation in post-traumatic OA (PTOA) mouse knee joints. **A** The volume of mineralised tissue was analysed with μCT imaging. Mineralisation in the joint space increased in the + 4 and +14 weeks post-trauma knee joints compared to healthy control knee joints. **B** 3D enlargement of the meniscus and ectopic mineralisation nodules showed mineralisation in the lateral and posterior compartments of the knee joint. Scale is 500 μm

The volume of mineralised tissue within the knee joint was quantified using μCT imaging. PTOA knee joints had increased mineralisation compared to healthy control joints at both 4 (33% increase, *p* < 0.01) and 14 weeks (46% increase, *p* < 0.001) (Fig. 2A). Mineralisation significantly increased at 14 weeks post-trauma compared to 4 weeks post-trauma (*p* = 0.02) (Fig. 2A), indicating progressive PTOA pathology. Furthermore, representative reconstructed 3D μCT images demonstrated mineralisation which was localised primarily to the lateral and posterior menisci and meniscal ligaments (Fig. 2B). These increases in mineralisation indicated structural changes to knee joint following trauma.

### Histological assessment of the post-trauma ACL revealed cellular hypertrophy and expression of chondrogenic markers

**Fig. 3:**
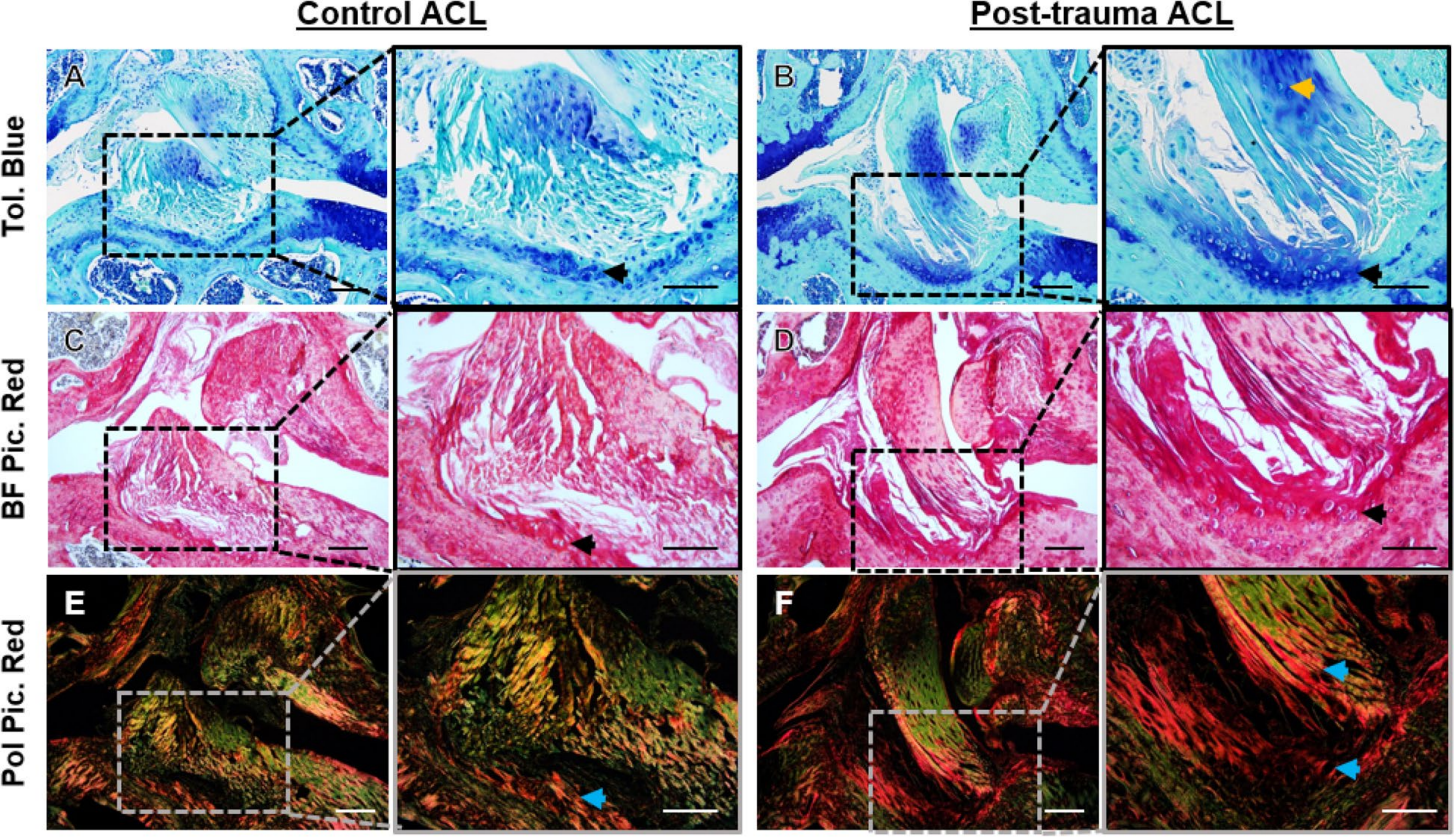
Representative histological staining in the anterior cruciate ligament (ACL) of control and post-trauma mouse knee joints. **A**, **B** Toluidine blue (Tol. Blue) staining of healthy and post-trauma ACL showed Tol. Blue staining in the mid-substance and tibial enthesis of the post-trauma ACL (black arrow). **C**, **D** Brightfield Picrosirius Red (BF Pic. Red) staining confirmed rounded-cell morphology in the tibial enthesis of the post-trauma ACL (black arrow). **E**, **F** Polarised Picrosirius Red (Pol Pic. Red) showed red collagen birefringence in the tibial enthesis extending towards the mid-substance of the post-trauma ACL (blue arrows). Scale is 100 μm and 50 μm for lower and higher magnification. Post-trauma ACL images are from + 4-weeks post-trauma

Histological staining of the knee joint revealed differences in the structural and cellular composition of the post-trauma ACLs. In the healthy control knee joints, the ACL mid-substance comprised of spindle-shaped cells with aligned fibres that stain only weakly with TB (Fig. 3A) and green collagen birefringence (Fig. 3E). In healthy ACL-tibial enthesis regions, there were fibrocartilaginous cells with rounded morphology surrounded by TB staining (Fig. 3A, black arrow) and red collagen birefringence (Fig. 3E). Following trauma, the ACL mid-substance had persistent TB staining and cells with rounded cell morphology (Fig. 3B, yellow arrow).

Furthermore, in the post-trauma ACL-tibial enthesis, there was a TB region encompassing cells with rounded morphology (Fig. 3B, black arrow), which corresponded with red collagen birefringence (Fig. 3F, blue arrows). Increases in TB staining and red collagen birefringence indicates a change in ECM composition and organisation following trauma.

**Fig. 4:**
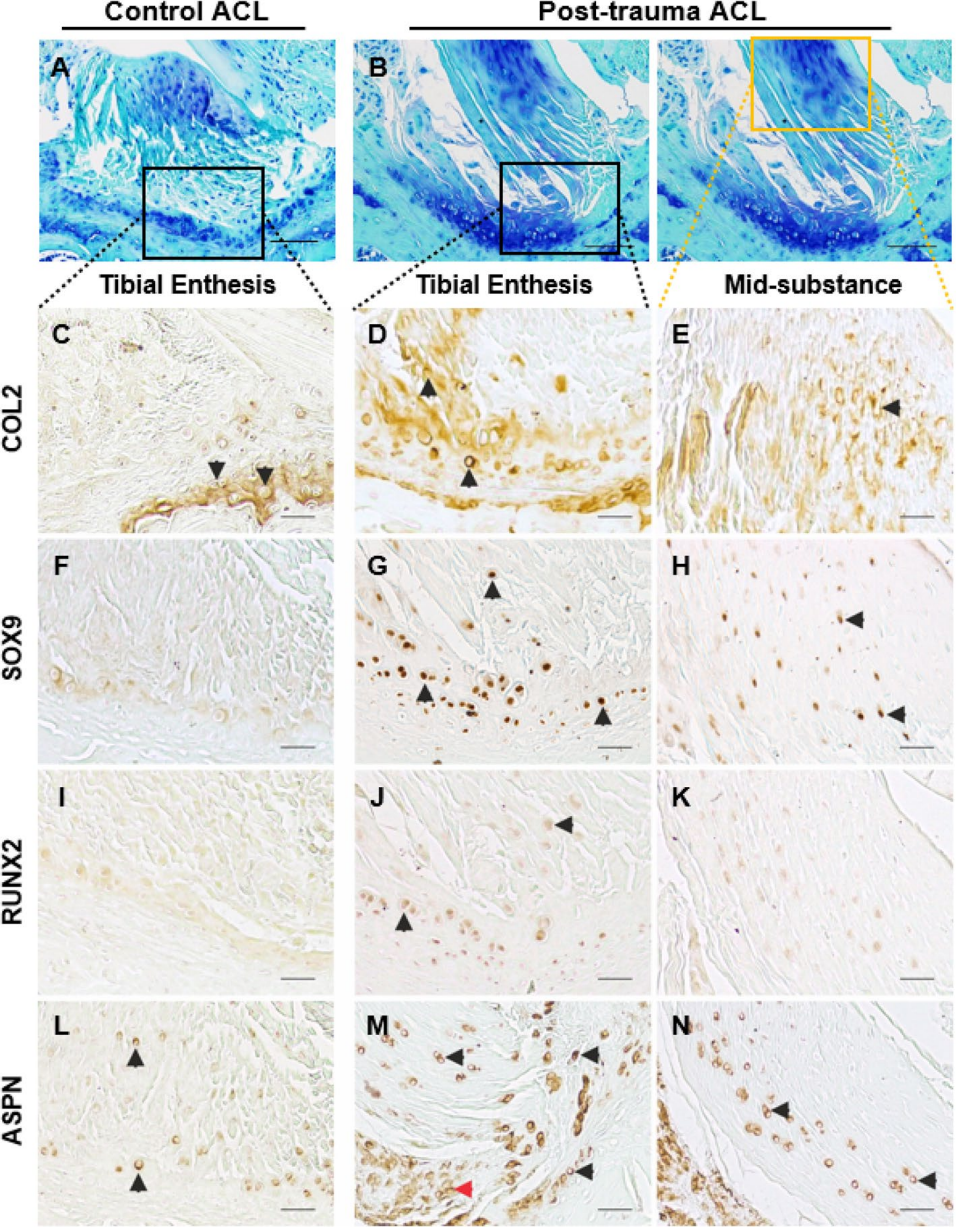
Representative marker expression in the anterior cruciate ligament (ACL) of control and post-trauma mouse knee joints with immunohistochemistry. **A**, **B** The ACLs of healthy (*n* = 6) (**A**) and post-trauma knee joints (*n* = 6) (**B**) were analysed across microanatomical regions. **C**–**E** Collagen type II (COL2) expression was present in the fibrocartilaginous tibial enthesis of the healthy ACL and in the mid-substance region of the post-trauma ACL (black arrows). **F**–**H** SOX9 chondrogenesis transcriptional factor expression was found in the tibial enthesis and mid-substance regions of the post-trauma ACL (black arrows). **I**–**K** RUNX2 hypertrophic transcriptional factor was expressed in the tibial enthesis region of the post-trauma ACL (black arrows). **L**–**N** Asporin (ASPN), a small leucine-rich proteoglycan, extended within the mid-substance of the post-trauma ACL. Scale is 25 μm for **C**–**N**, and 50 μm for **A**–**B**. Post-trauma ACL images are from + 4 weeks post-trauma

Staining for specific matrix and cellular markers via immunohistochemistry confirmed modifications to ECM composition following trauma. Collagen type II (COL2) was present, as expected, in the fibrocartilaginous region of the ACL-tibial enthesis of the healthy control knee joint (Fig. 4C, black arrow). COL2 expanded towards to mid-substance region in post-trauma knees (Fig. 4D, E, black arrow). Cellular changes included expression of chondrogenic marker SOX9, hypertrophic marker RUNX2 and small-leucine rich proteoglycan ASPN. Both SOX9 and RUNX2 expressions were faint in the healthy control ACL-tibial enthesis (Fig. 4F, I) but were clearly expressed in post-trauma ligament cells at the ACL-tibial enthesis (Fig. 4G, J) and ACL mid-substance (Fig. 4H, K). Similarly, pericellular ASPN expression was limited in the healthy control knee joint in the ACL-tibial enthesis (Fig. 4L) but was seen throughout the ACL (black arrows) and in surrounding regions (red arrow) (Fig. 4M, N) post-trauma.

Immunohistochemistry confirmed changes to the ECM composition and cellular phenotype in the post-trauma ACL, with suggestions of early endochondral ossification pathways being activated (SOX9, COL2, ASPN).

### Viscoelastic stiffness and strain rate sensitivity decreased, and relaxation increased in the post-trauma ACL

**Fig. 5:**
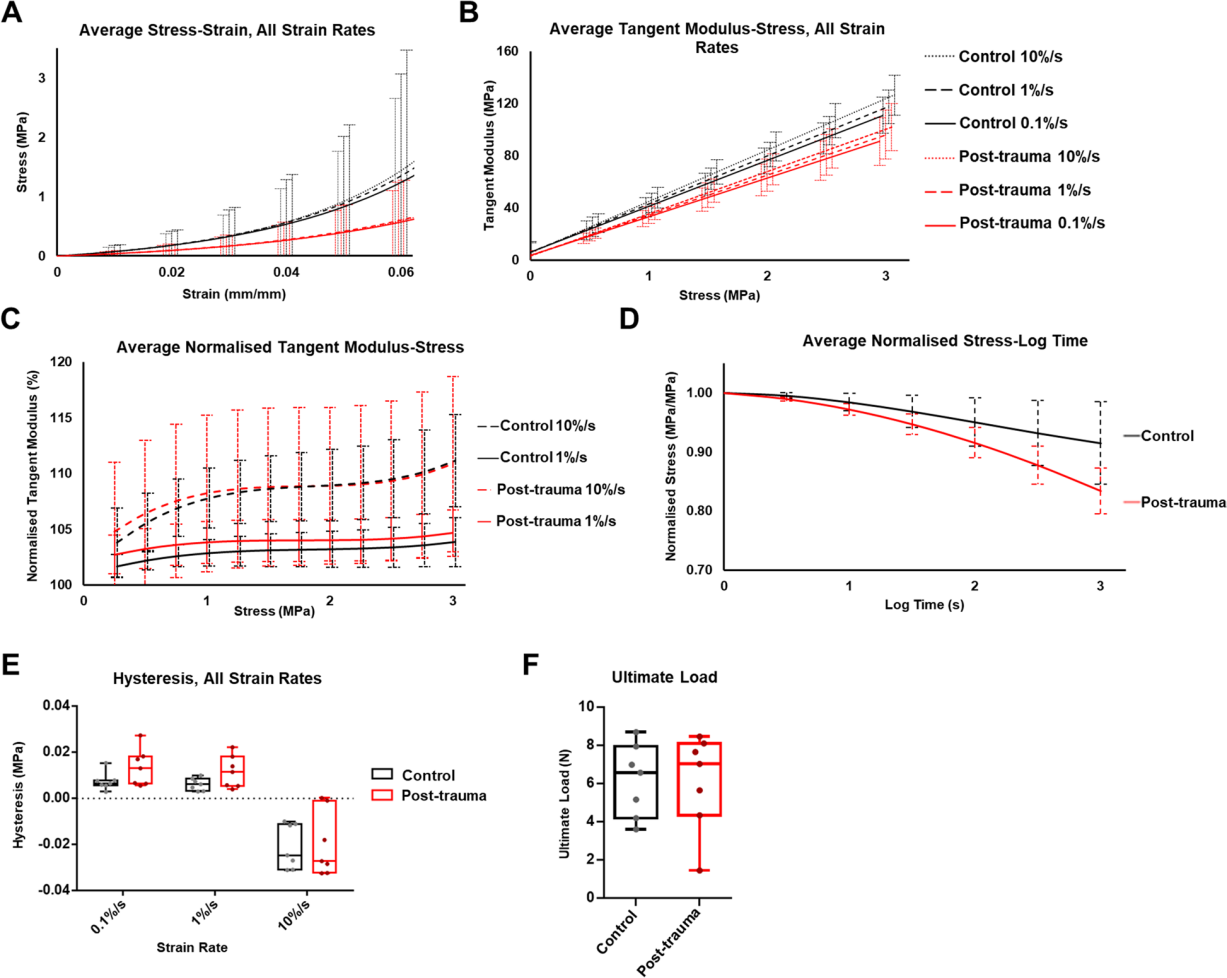
Mechanical and viscoelastic properties of the anterior cruciate ligament (ACL) of control and post-trauma mouse knee joints. **A**, **B** Average stress-strain and tangent modulus-stress curves showed a decrease in stiffness at all strain rates for the post-trauma ACLs compared to healthy ACLs. **C** Normalised tangent modulus-stress curves (normalised from the 0.1%/s strain rate curve) compared differences in 1%/s and 10%/s strain rates, which were statistically significant in the control ACLs (*p* < 0.01) but not in the post-trauma ACLs (0 = 0.07), suggesting a lack of strain rate sensitivity in the post-trauma ACL. **D** Stress-relaxation curves showed lower normalised stress in the post-trauma ACLs. **E** Hysteresis curves at different strain rates showed no statistical difference between control and post-trauma (0.1%/s: *p* = 0.09, 1%/s: *p* = 0.08, 10%/s: *p* = 0.88). **F** Ultimate load at failure showed no statistical difference between control and post-trauma ACLs. Control = black, Post-trauma = red. Post-trauma ACLs are from + 6 weeks post-trauma

Mechanical properties of mouse ACLs following trauma were assessed and compared to healthy controls. Stress-strain plots at strain rates of 0.1%/s, 1%/s and 10%/s showed exponential viscoelastic behaviour in healthy ACLs (Fig. 5A). Post-trauma ACLs also demonstrated similar viscoelastic behaviour. At 0.03 mm/mm strain, the midpoint, stress in the post-trauma ACLs was not significantly different from the control ACLs at any strain rate (*p* = 0.19 for 0.1%/s, *p* = 0.20 for 1%/s, *p* = 0.21 for 10%/s) (Fig. 5A). However, the tangent modulus-stress plots, which provide stiffness estimates at different stress levels, demonstrated reductions in the tangent modulus in post-trauma ACLs. At 2 MPa stress (the midpoint), the tangent modulus was significantly decreased by 20% at 0.1%/s strain rate relative to control ACLs (*p* = 0.03), 20% at 1%/s strain rate (*p* = 0.02) and 21% at 10%/s strain rate (*p* = 0.02) (Fig. 5B). These results suggest a decrease in ACL stiffness in the toe-region of the post-trauma knee joint stress-strain behaviour compared to the healthy ACL.

Strain-rate sensitivity was analysed in the stress-strain, tangent modulus-stress, and normalised tangent modulus-stress curves. In the stress-strain curves, at 0.03 mm/mm strain, no significant differences were found between different strain rates in the control ACLs (*p* = 0.17) and the post-trauma ACLs (*p* = 0.28) (Fig. 5A). In the tangent modulus-stress curves, strain-rate differences were statistically significant at 2 MPa stress for both the control (*p* < 0.01) and the post-trauma ACLs (*p* < 0.01) (Fig. 5B), suggestive of strain rate sensitivity in both ACL groups. Post hoc analysis comparing specific strain rates revealed differences at higher strain rates. In the post-trauma ACLs, at 2 MPa of stress, there was no statistical differences between the 0.1%/s and 1%/s strain rates and the 1%/s and 10%/s strain rates (*p* = 0.15 for both). In the control ACLs, at 2 MPa, post hoc analysis showed statistical differences between all strain rates (*p* < 0.01 for all). This would suggest a decrease in strain rate sensitivity in the post-trauma ACLs. Normalised tangent modulus-stress curves confirmed these findings, as strain rate differences between 1%/s and 10%/s led to significant differences at 2 MPa of stress in the control ACLs (*p* < 0.01) but not in the post-trauma ACLs (*p* = 0.07) (Fig. 5C). This is suggestive of a decrease in strain rate sensitivity in the post-trauma ACLs at higher strain rates.

The normalised stress was lower throughout the stress-relaxation response in the post-trauma ACLs compared to the healthy control ACLs (*p* = 0.04) (Fig. 5D). This was indicative of an increased relaxation response of the post-trauma ACLs compared to the healthy ACLs.

When comparing the hysteresis response between the control and post-trauma ACLs, there were no statistical differences at 0.1%/s (*p* = 0.09), 1%/s (*p* = 0.08) and 10%/s (*p* = 0.88) (Fig. 5E). Furthermore, negative hysteresis was found in the 10%/s strain rate for both the control and post-trauma ACLs (Fig. 5E). Therefore, changes in viscoelastic behaviour were seen in the strain rate sensitivity and stress-relaxation but not hysteresis.

The material strength of the ACL was also measured with the ultimate load to failure, which was similar in the healthy control and post-trauma ACLs. The ultimate load was 6.17 ± 1.91 N and 6.10 ± 2.51 N for the control and post-trauma ACLs respectively (*p* = 0.96) (Fig. 5F). This would imply similar material strengths of the control and post-trauma ACLs, despite differences in viscoelastic properties.

Overall, viscoelastic properties of the post-trauma ACL demonstrated a decrease in stiffness, strain rate sensitivity and normalised stress during stress-relaxation, which reinforces our hypothesis that viscoelastic properties of the ACL are modified post-trauma.

## Discussion

This study found that repetitive trauma in the knee joint resulted in changes in the ECM components and viscoelastic mechanical properties of the ACL. These findings confirm that ACL pathologies result in functional anomalies, which could contribute to knee stability and the progression of PTOA. Specifically, we have found that structural changes post-trauma, particularly proteoglycan and COL2 deposition, were associated with a decrease in ligament stiffness, a decrease in strain rate sensitivity and an increased relaxation behaviour during stress-relaxation. Changes in the strain rate sensitivity of the ACL following knee trauma is, to our knowledge, a novel finding and suggests a decrease in stiffness at higher strain rates.

Increases in joint space mineralisation measured using μCT was specific to the lateral and posterior compartments, which correlated with the location of PTOA development in this model [22]. An increase in mineralisation in knee joints has been previously shown in other spontaneous and post-traumatic OA mouse models using the same method [3]. These studies suggest a strong relationship between articular cartilage degeneration and pathological joint tissue mineralisation and support the use of μCT imaging as a measure of OA disease progression. Further studies will determine the cellular basis of this pathological joint mineralisation, including in ligaments, and whether these could be potential disease targets.

Changes in the ACL matrix composition during post-trauma included increased TB staining, indicative of proteoglycan deposition [36], red collagen birefringence and COL2 deposition. Red collagen birefringence suggests thicker collagen organisation at the ACL-tibial enthesis [27, 37]. Interestingly, these changes in collagen organisation did not result in stiffer viscoelastic mechanical properties of the ACL. Changes in the ECM were seen at the tibial enthesis and mid-substance of the ACL. Changes to ACL microanatomy reflect the history of trauma forces and are clinically important for the development of better ACL grafts, for future targeted therapeutics and to understand the development of different types of ACL tears (mid-substance vs avulsion fracture).

Viscoelastic behaviour of the post-trauma ACLs showed changes in the tangent modulus, strain rate sensitivity and stress-relaxation properties. These changes included a significant decrease in the tangent modulus, implying a decrease in viscoelastic stiffness. It is likely that ECM components are driving this viscoelastic change, including collagen type II deposition. Collagen type II molecules have a lower elastic modulus (stiffness) than collagen type I molecules [38] and therefore could result in a decrease in tangent modulus. However, the role of COL2 in viscoelastic behaviour needs to be further assessed, including whether COL2 is stretched in the toe region and how it interacts with the surrounding matrix.

Stress-relaxation curves decreased in post-trauma ACLs, indicating an increase in relaxation. Changes in viscoelastic stress-relaxation has previously been associated with glycosaminoglycan and proteoglycan depletion in tendons [9, 39]. Proteoglycan depletion is not likely in the post-trauma ligaments which had an increase in TB staining. This could suggest that a different matrix component is driving changes in stress-relaxation.

Increases in relaxation the ACL stress-relaxation behaviour in human OA and rheumatoid knees have been reported previously [17], reinforcing similar viscoelastic pathology between human OA and mouse PTOA. However, the ACL of OA patients also had a decrease in ultimate load [17], not seen in the post-trauma ACLs in mice. These differences could arise from differences in between post-trauma and age-related human OA.

Changes in strain rate sensitivity in ACLs post-trauma have not been previously reported. Our study shows for the first time a decrease in the strain rate sensitivity of the higher strain rates (1% and 10%/s) which suggests a decrease in the ECM stiffness at higher strain rates. Strain rate sensitivity is another viscoelastic property associated with proteoglycan composition. A reduction in strain rate sensitivity has been reported in decorin and biglycan depleted tendons [9, 10, 40]. Computational modelling has also supported the interfibrillar viscoelastic role of proteoglycans [7]. However, collagen fibrils have greater elongation at higher strain rates [41], and theoretically could play a more important role at higher strain rates. Our study also reported ASPN expression in diseased ACLs; however, ASPN lacks glycosaminoglycan chains [42] associated with increased viscosity [43] and therefore is unlikely to play a role.

Previous studies have shown that tendon fibroblasts respond to mechanical stimuli by adjusting ECM stiffness [44, 45]; therefore, it is possible that ligament fibroblasts are driving ECM and viscoelastic changes. However, the mechanotransduction pathways involved are unknown. This study showed SOX9, RUNX2 and COL2 expression in the ACL of the post-trauma knee joint, in the same area where we identified cells with rounded morphology. SOX9, COL2 and rounded-cell morphology were previously seen in ligaments of spontaneous OA mouse models [3] and in ligaments of age-related human OA [5], indicating similar pathology post-trauma and in age-related OA. These are known markers of chondrogenesis and hypertrophy, crucial for endochondral ossification. ASPN, a small-leucine rich proteoglycan, is a marker previously seen localised to ligament cells and expressed in tendons [46]. ASPN is also found in the cartilage of human OA patients inhibiting TGF-β-induced expression of the *Col2a1* gene [47] and is a negative regulator of periodontal ligament mineralisation by interacting directly with BMP-2 [48]. The effects of ASPN in post-trauma ligaments remains unknown.

It remains unclear whether ligament viscoelastic changes contribute to PTOA progression. COL2 in tendons has been shown to increase following compressive load [49, 50] and is likely to be a direct result of the non-invasive loading regime. However, ACL mechanics and OA are closely related including in spontaneous OA models with no history of trauma [3, 19, 20]. Matrix viscoelastic properties have been shown to regulate pathology, including stress-relaxation properties which can regulate scaffold remodelling and bone formation [51]. Therefore, it is possible viscoelastic changes in injured ligaments could drive chondrogenesis or mineralisation.

Limitations of this study include the mechanical testing of the mouse ACL. Stress and strain were calculated with the assumption of similar ACL length and CSA within groups. Most studies on mouse ACL mechanics measured the CSA and length for each sample [11, 12, 20]. This could account for the deviation seen in the stress-strain curves and negative hysteresis. Negative hysteresis has been reported in human tendons [52, 53] and attributed to inaccuracies. However, negative hysteresis was only measured at high strain rates, similar to what has been reported in the human Achilles tendon [52]. Past studies in human skin have attributed negative hysteresis to varying viscoelastic properties of different skin layers [54]. Therefore, negative hysteresis could be a result of differences in ACL bundles or fascicles.

Other limitations include the lack of ligament-specific and ACL-specific fibroblast and matrix markers. Markers in healthy adult ligaments are not well characterised and future research should focus on validating markers across microanatomical regions as this will benefit the understanding of ligament disease pathways. In addition, future research on the ACL post-trauma should explore the role of hypertrophic ligament cells and their signalling pathways to identify the underlying cellular processes and confirm if matrix remodelling can lead to endochondral ossification or other types of fibrotic healing.

## Conclusions

Overall, this research showed that ligament matrix composition and viscoelastic behaviour change following knee trauma, potentially driven by cellular expression of chondrogenic and hypertrophic markers (COL2, SOX9, RUNX2). It remains unknown how these changes affect PTOA progression. Furthermore, we have confirmed our hypothesis that structural changes in the ACL post-trauma resulted in a reduction in viscoelastic stiffness and other viscoelastic properties. This confirms that the ligament biomechanical microenvironment is altered following injury. Understanding the relationship between the structure and mechanical function is necessary to better understand the underlying post-trauma pathways. In the future, targeting these molecular and cellular changes might improve ligament function and prevent additional PTOA progression.

## Supplementary Information

**Additional file 1: Supplemental Table 1**. Angles of in vivo knee joint flexion and gait speed as determined by biplanar radiography. The average minimum (Min) and maximum (Max) knee flexion was calculated in degrees for both the left and right knee joint. Knee flexion ranged from 56.5 to 100.8° in the left knee, and 55.6 and 100.5° in the right knee joint. Relative standard deviation (RSD) ranged from 6.7% to 13.3%. **Supplemental Table 2**. Ex vivo knee joint anterior cruciate ligament (ACL) measurements. Measurements of the ACL were taken from healthy and post-trauma knee joints and included ACL length and ACL cross-sectional area (CSA). For each measurement relative standard deviation (RSD) and percent different (%diff) was calculated. CSA and length of control and post-trauma ACLs were not significantly different (*p*=0.6 for CSA, and *p*=0.08 for length). **Supplemental Figure 1**. In vivo knee flexion measurements using biplanar X-ray. A) X-ray images were imported to XMALab software (Brown University, USA) where each anatomical point (Pt.) was tracked and numbered (1 to 7). B) Anatomical points tracked were the following: left hip (1, LHIP), left knee (2, LKNEE), left ankle (3, LANK), right hip (4, RHIP), right knee (5, RKNEE), right ankle (6, RANK), and occiput (7, OCCI). C) A 3D model of gait for the left and right hind-legs was created from which physiological knee range of motion during gait was calculated [11, 30, 34, 35, 55].

## Data Availability

The datasets used in this study is available from the corresponding author on reasonable request.

## Abbreviations

ACL: Anterior cruciate ligament
ASPN: Asporin
BMP: Bone morphogenetic proteins
COL2: Collagen type II
CSA: Cross-sectional area
ECM: Extracellular matrix
OA: Osteoarthritis
PTOA: Post-traumatic osteoarthritis
RUNX2: Runt-related transcription factor 2
SOX9: SRY-box 9
TB: Toluidine blue
TGF-β: Transforming growth factor β
μCT: Micro-computed tomography
