# Supplementary material for: The anterior cruciate ligament in murine post-traumatic osteoarthritis: markers and mechanics
Source: Arthritis Res Ther. 2022 May 30;24:128. doi: 10.1186/s13075-022-02798-7 (PMC9150328; doi:10.1186/s13075-022-02798-7)
Supplement: Supplementary file 1 — Additional file 1: Supplemental Table 1. Angles of in vivo knee joint flexion and gait speed as determined by biplanar radiography. The average minimum (Min) and maximum (Max) knee flexion was calculated in degrees for both the left and right knee joint. Knee flexion ranged from 56.5 to 100.8° in the left knee, and 55.6 and 100.5° in the right knee joint. Relative standard deviation (RSD) ranged from 6.7% to 13.3%. Supplemental Table 2. Ex vivo knee joint anterior cruciate ligament (ACL) measurements. Measurements of the ACL were taken from healthy and post-trauma knee joints and included ACL length and ACL cross-sectional area (CSA). For each measurement relative standard deviation (RSD) and percent different (%diff) was calculated. CSA and length of control and post-trauma ACLs were not significantly different (p=0.6 for CSA, and p=0.08 for length). Supplemental Figure 1. In vivo knee flexion measurements using biplanar X-ray. A) X-ray images were imported to XMALab software (Brown University, USA) where each anatomical point (Pt.) was tracked and numbered (1 to 7). B) Anatomical points tracked were the following: left hip (1, LHIP), left knee (2, LKNEE), left ankle (3, LANK), right hip (4, RHIP), right knee (5, RKNEE), right ankle (6, RANK), and occiput (7, OCCI). C) A 3D model of gait for the left and right hind-legs was created from which physiological knee range of motion during gait was calculated [11, 30, 34, 35, 55]. [file 13075_2022_2798_MOESM1_ESM.docx]

# 10 Supplementary Information

## 10.1 ACL histological staining

TB staining buffer was 0.1% TB in 0.1M solution of acetate buffer, pH 5.6. Slides were stained with TB buffer for 15 minutes and counterstained with 0.2% fast green for 5 seconds and then rinsed with acetone twice for 10 seconds. For picrosirius red, sections were stained in Weigert’s haematoxylin for 8 minutes then stained with picrosirius red solution (0.1% w/v sirius red in saturated aqueous picric acid) for one hour and then washed with 0.5% v/v glacial acetic acid in distilled water for 3 minutes. Slides were then dehydrated, mounted and imaged with an Olympus microscope with an adjustable polariser (Olympus BX60).

## 10.2 ACL histological immunostaining

For COL2 immunostaining, antigen retrieval was applied with pepsin (3mg/mL in 0.02M HCl) for 45 minutes at 37ᵒC. Slides were then blocked for endogenous peroxidase with 0.3% hydrogen peroxide (Sigma, 15 minutes), for endogenous Avidin/Biotin binding (Vector Labs, SP2001; 15 minutes each), and for non-specific binding (COL2: Mouse-on-Mouse Kit, Vector Labs, BMK-2202; SOX9, RUNX2, ASPN: 10% v/v goat serum). Primary antibodies were incubated overnight at 4°C and included COL2 (1/100), SOX9 (1/1000), RUNX2 (1/1000), ASPN (1/400). Negative controls included a mouse IgG (2µg/mL, Sigma) or rabbit IgG (1µg/mL, Vector Labs). Biotinylated secondary antibody (Vector Labs; 1/200 for 1h) and then Vectastain solution (Vector Labs) were applied. Stains were developed with 3,3′-Diaminobenzidine (Vector Labs), dehydrated, mounted and imaged with a microscope (Zeiss).

## 10.3 Calculations for viscoelastic properties of the mouse ACL

For stress-strain behaviour, the average ACL length and CSA measurements for the post-trauma and control ACL (Section 3.4 and Sup. Table 2) were used to calculate the stress and strain using Equations 1 and 2, respectively. The resulting stress-strain curve was then fitted to an exponential curve suitable for ligament stress-strain behaviour [55]. From the exponential best-fit curve for each strain rate, the tangent modulus (gradient of the stress-strain curve) at each stress or strain was derived using Equation 3.

|  | $\sigma=\frac{F}{CSA}$ | (Equation 1) |
| --- | --- | --- |

where σ is stress (MPa), F is the applied force (N) and CSA (mm^2^) is the estimated cross-sectional area.

|  | $\varepsilon=\frac{\Delta L}{L_{0}}$ | (Equation 2) |
| --- | --- | --- |

where ε is strain (mm/mm), ∆L is the corresponding change in elongation (mm) (∆L = L_t_ – L_0_), L_0_ is the initial estimated ligament length (mm), and L_t_ is the deformed length of the ligament (mm).

|  | $E_{tan}= \frac{\Delta\sigma}{\Delta\varepsilon}$ | (Equation 3) |
| --- | --- | --- |

where E_tan_ is tangent modulus (MPa).

To determine the sensitivity of stress-strain behaviour to changes in strain rate, estimations of the stress-strain behaviour and tangent modulus were repeated for all the different strain rates (0.1%/s, 1%/s, 10%/s). Tangent modulus values were normalised to the 0.1%/s strain rate tangent modulus of the same sample using Equation 4.

|  | $E_{norm}(\sigma)= \frac{E_{s}(\sigma)}{E_{0}(\sigma)}x 100$ | (Equation 4) |
| --- | --- | --- |

where E_norm_(σ) is the normalised tangent modulus (%) at a specific stress, E_tan_(σ) is the tangent modulus at 1%/s or 10%/s strain rates at the corresponding stress, and E_0_(σ) is the tangent modulus at 0.1%/s strain rate at the corresponding stress.

Stress-relaxation behaviour measured the stress degradation over time while maintaining a strain of 5%. Stress was normalised by the peak stress at t=0 (when 5% strain was reached) [11] using Equation 5. The stress-relaxation curve was fitted into a polynomial equation to determine the average normalised stress for all samples.

|  | $\sigma_{norm}\left( t \right)= \frac{\sigma_{t}}{\sigma_{0}}$ | (Equation 5) |
| --- | --- | --- |

where σ_norm_(t) is the normalised stress (MPa/MPa) over log time, σ_o_ the peak stress at t=0 and σ_t_ is the stress behaviour over log time.

Hysteresis was calculated from the load-elongation data to measure the area between the load and unload cycles which correlates to the energy dissipated due to material viscosity. Hysteresis was measured using numerical integration (trapezoidal rule) as described previously [34, 35].

## *10.4 In vivo* gait analysis of knee flexion

*In vivo* gait analysis tests to determine the physiological range of motion in the knee joint was conducted on healthy B6CBAF1 mice (n=4) (Charles River). Biplanar X-ray was recorded using two independent 60 kW Epsilon X-ray generators (EMD Technologies, CA), 16 inch image intensifier tubes (Thales, FR), X-Ray Tubes (Varian, USA) and Phantom Miro M120 video cameras (Vision Research, USA). X-ray images of gait (Sup. Fig. 1A) were analysed using XMALab Software (Brown University, USA) and MatLab (MathWorks, R2018a Version 9.4, USA). For each mouse, two gait trials were analysed. X-ray generator pulsed radiographic exposures at 120 frames per second, and video was recorded for approximately 3 seconds. Video files were corrected for distortion, calibrated, and digitised in XMALab software. Mouse gait was tracked at every frame at the following points: left hip, left knee, left ankle, right hip, right knee, right ankle, and occiput (Sup. Fig. 1B). The resulting locations of each point were exported and in MatLab a 3D model was created (Sup. Fig. 1C) and knee speed and knee joint flexion was measured at every frame. The results from this gait analysis of physiological knee flexion were used to determine the ideal physiological angle for mechanical testing of the femur-ACL-tibia complex.


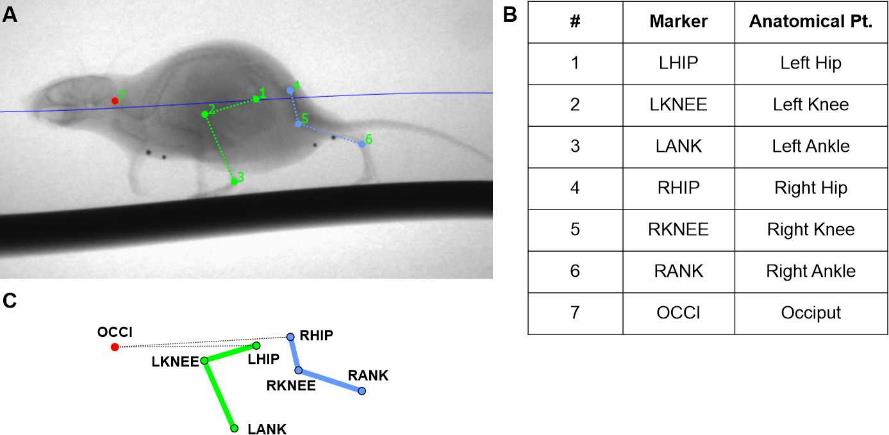


**Supplemental Figure 1: In vivo knee flexion measurements using biplanar X-ray.**

A) X-ray images were imported to XMALab software (Brown University, USA) where each anatomical point (Pt.) was tracked and numbered (1 to 7). B) Anatomical points tracked were the following: left hip (1, LHIP), left knee (2, LKNEE), left ankle (3, LANK), right hip (4, RHIP), right knee (5, RKNEE), right ankle (6, RANK), and occiput (7, OCCI). C) A 3D model of gait for the left and right hind-legs was created from which physiological knee range of motion during gait was calculated.

Maximum knee flexion was 100.8° in the left knee joints and 100.5° in the right knee joint (Sup. Table 1). Minimum knee flexion was 56.5° and 55.6° in the left and right knee respectively (Sup. Table 1). Maximum and minimum angles of knee flexion in both the left and right knee joints of the B6CBAF1 (n=4) mice were similar. This data suggests that the in vivo physiological range of knee flexion during gait was between 55-100°. In order to remain within the physiological range, mechanical viscoelastic and material testing of the ACL was performed at 90° of knee flexion, because it allowed for uniform tensile testing along the axis of the ligament, similar to a previous study by Warden et al [30].

**Supplemental Table 1. Angles of in vivo knee joint flexion and gait speed as determined by biplanar radiography.**

|  | **Degrees (°)** | | | | **Gait speed** | |
| --- | --- | --- | --- | --- | --- | --- |
|  | **Min** | **Max** | **Min** | **Max** | **(mm/s)** | |
|  | **Left Knee** | | **Right Knee** | | **Left knee** | **Right knee** |
| Avg. | 56.5 | 100.8 | 55.6 | 100.5 | 135.6 | 138.7 |
| RSD (%) | 8.8 | 13.3 | 6.7 | 11.5 | 24.3 | 19.1 |

The average minimum (Min) and maximum (Max) knee flexion was calculated in degrees for both the left and right knee joint. Knee flexion ranged from 56.5 to 100.8° in the left knee, and 55.6 and 100.5° in the right knee joint. Relative standard deviation (RSD) ranged from 6.7% to 13.3%.

## 10.5 Anterior cruciate ligament (ACL) measurements

ACL length and CSA were measured using µCT. The ACL length of the control and post-trauma knee joint ACLs was 1.14 ±0.10 mm and 1.09 ±0.09 mm respectively and were not significantly different (p=0.08). The ACL CSA was 0.105 ±0.017 mm^2^ for the control knee joints and 0.109 ±0.017 mm^2^ for the post-trauma knee joints and were not significantly different (p=0.6) (Sup. Table 2). The ACL measurements for each mouse group were used to calculate their viscoelastic properties.

**Supplemental Table 2. Ex vivo knee joint anterior cruciate ligament (ACL) measurements.**


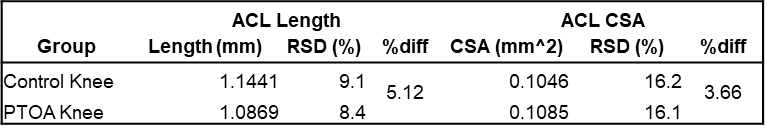


Measurements of the ACL were taken from healthy and post-trauma knee joints and included ACL length and ACL cross-sectional area (CSA). For each measurement relative standard deviation (RSD) and percent different (%diff) was calculated. CSA and length of control and post-trauma ACLs were not significantly different (p=0.6 for CSA, and p=0.08 for length).
